# Tendinopathie achilléenne compliquée d´une rupture du tendon d´Achille consécutives à une automédication par la ciprofloxacine chez une hémodialysée: à propos d´un cas

**DOI:** 10.11604/pamj.2021.38.312.9269

**Published:** 2021-03-29

**Authors:** Gaël Clovis Gassongo Koumou, Kevin Parfait Bienvenu Bouhelo Pam, Mohamed Arrayhani, Tarik Sqalli Houssaini, Abdelmajid El Mrini

**Affiliations:** 1 Service de Néphrologie, Centre Hospitalier Universitaire Hassan II, Fès, Maroc,; 2 Service de Traumatologie-Orthopédie B4, Centre Hospitalier Universitaire Hassan II, Fès, Maroc,; 3 Equipe de Recherche *Renal Exploration and Investigations in Nephrology (REIN)*, Faculté de Médecine et de Pharmacie de Fès, Fès, Maroc

**Keywords:** Tendinopathie achilléenne, rupture du tendon d´Achille, effets secondaires, ciprofloxacine, rapport de cas, Achilles tendinopathy, rupture of the Achilles tendon, side effects, ciprofloxacin, about a case

## Abstract

L´objectif de ce travail était de rappeler aux praticiens cette complication des fluoroquinolones, la tendinopathie. Il s´agit d´un effet secondaire rare mais, qui peut être source de handicap fonctionnel. Une femme de 79 ans, hémodialysée depuis 11 années avait présenté une douleur soudaine de la cheville gauche et une impotence fonctionnelle relative du membre homolatéral au 5^e^ jour d´une automédication par la ciprofloxacine. Elle avait comme comorbidités, une gonarthrose chronique, une hyperparathyroïdie secondaire et une cardiopathie ischémique. Le diagnostic de tendinopathie achilléenne bilatérale et d´une rupture achilléenne gauche étaient retenus sur la base des arguments cliniques, confortés par l´échographie achilléenne. La responsabilité de la ciprofloxacine était évaluée à l´aide de la méthode française d´imputabilité des effets inattendus ou toxiques des médicaments. La prise en charge était chirurgicale suivie d´une rééducation fonctionnelle avec une évolution satisfaisante. La fréquence des tendinopathies liées aux fluoroquinolones varie de 15 à 20 accidents pour 100 000 sujets traités dont un tiers de cas sont compliqués de rupture tendineuse. L´incidence est liée à l´âge, avec un âge de prédilection > 60 ans impliquant le vieillissement tissulaire. La péfloxacine et la ciprofloxacine sont les molécules les plus incriminées. Le délai d´apparition des symptômes noté à cinq jours dans cette observation corrobore avec la littérature. Nous avons retrouvé certains facteurs favorisants connus à savoir: insuffisance rénale chronique, dialyse, corticothérapie, hypolipémiant (statine). La présentation clinique est classique, suffisante pour poser le diagnostic. La localisation est majoritairement achilléenne. Elle est bilatérale dans 40 à 66%. La rupture tendineuse est la principale complication. La prise en charge est chirurgicale. Elle permet de rétablir l´anatomie et de prévenir le handicap fonctionnel préjudiciable. Nous rapportons un effet secondaire rare mais, potentiellement grave, lié aux fluoroquinolones et, qui expose à un handicap fonctionnel. L´âge avancé, l´insuffisance rénale chronique, l´hémodialyse chronique, la prise concomitante de statine et de corticoïde sont des facteurs traditionnels favorisants retrouvés ici. Les hémodialysés constituent une population à risque pour qui une surveillance même à distance d´un traitement à base de ces molécules revêt un intérêt non négligeable.

## Abstract (en)

This study aims to remind clinicians of fluoroquinolone-related tendinopathies. They are rare side effects, but which can result in functional disability. We report the case of a 79-year-old woman with a 11-year history of haemodialysis who had sudden left ankle pain and functional impairment in the ipsilateral member on day 5^th^ after self-medication with ciprofloxacin. Comorbidities included chronic gonarthrosis, secondary hyperparathyroidism and ischemic heart disease. The diagnosis of bilateral Achilles tendinopathy and rupture of the left Achilles tendon was retained due to clinical features and confirmed by ultrasound of ankles. Ciprofloxacin-associated tendon rupture was evaluated using the French method of accountability for drug unexpected side effects or toxicity. Tendon rupture management was based on surgery followed by functional rehabilitation program with satisfactory outcome. The frequency of fluoroquinolone-related tendinopathies ranges from 15 to 20 accidents per 100,000 subjects treated, a third of whom are complicated by tendon rupture. Incidence is related to age, affecting mainly people > 60 years and involving tissular aging. Pefloxacin and ciprofloxacin are the most offending molecules. In our study, the delay in the onset of symptoms on day 5 after self-medication was consistent with literature. We detected some common contributing factors including chronic renal failure, hemodialysis and the assumption of statins and corticosteroids. Fluoroquinolone-related tendinopathies are characterized by common clinical features which allow diagnosis. They mostly affect Achilles tendon. They are bilateral in 40-66% of cases. Tendon rupture is the main complication. Management is based on surgery. It allows to restore anatomy and to prevent detrimental functional disability. We here report a rare but potentially serious fluoroquinolones-related side effect, exposing the patient to the risk of functional disability. Advanced age, chronic renal failure, chronic haemodialysis, concomitant use of statins and corticosteroids are common contributing factors confirmed in this study. Hemodialysis patients constitute a population at risk; hence the importance of remote monitoring after treatment with these molecules.

## Introduction

Les fluoroquinolones sont des agents antibactériens caractérisés par une bonne disponibilité, une excellente diffusion tissulaire et un spectre élargi, utilisés pour la première fois en 1980. Leur accessibilité est de nos jours rendue facile avec l´essor des formes génériques. On leur décrit des effets secondaires multiples parmi lesquels les atteintes tendineuses ou tendinopathies touchant le plus souvent les tendons achilléens. Les premiers cas remontent à 1983 secondaires à la norfloxacine [1]. Les cas relatifs à la ciprofloxacine furent publiés pour la première fois en 1988 [2]. Il s´agit d´un effet secondaire de classe. Le principal risque est la rupture tendineuse. Plusieurs facteurs favorisants sont associés à cette complication: l´âge, l´insuffisance rénale, l´hémodialyse, la corticothérapie [1]. Il nous a paru opportun de faire part de cette observation afin de rappeler aux praticiens cette complication rare, mais qui peut être source de handicap fonctionnel.

## Patient et observation

**Figure 1:**
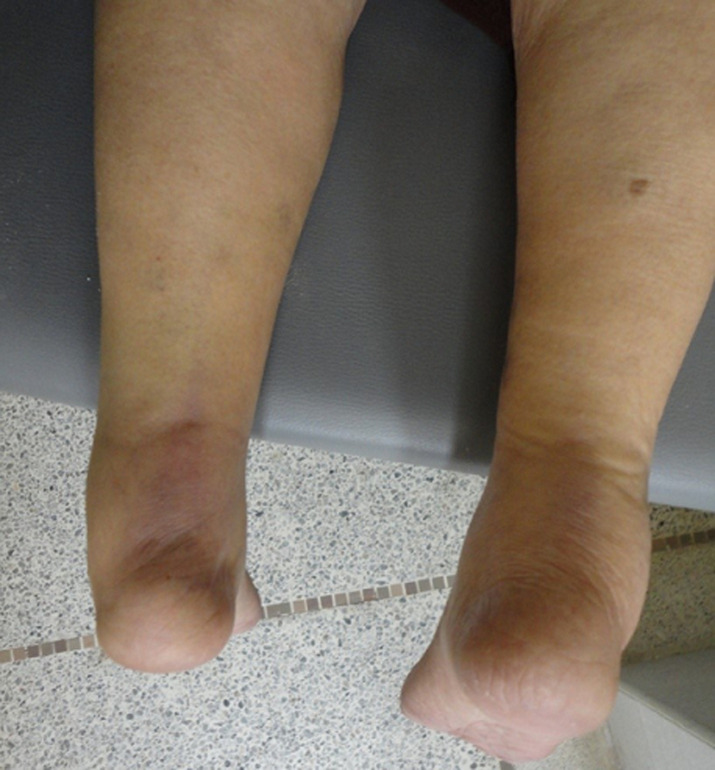
diagnostic clinique de la rupture du tendon d´Achille par l´inspection: la patiente installée en décubitus ventral, les pieds dépassant la table d´examen, l´on note une tuméfaction bilatérale du relief achilléen et une perte d´équinisme physiologique du pied gauche témoin de la rupture tendineuse

Une femme de 79 ans, hémodialysée depuis 11 années avait présenté une douleur d´installation soudaine, localisée à la cheville gauche et associée à une impotence fonctionnelle relative du membre inférieur homolatéral. La symptomatologie était apparue au cinquième jour, d´une automédication par de la ciprofloxacine per os 500 mg deux fois par jour, pour un syndrome grippal. Elle avait comme comorbidités, une arthrose chronique des deux genoux datant de deux ans, traitée par des infiltrations trimestrielles de méthylprednisolone 80 mg; une hyperparathyroïdie secondaire et une cardiopathie ischémique découverte un an auparavant. Dans son traitement, on retrouvait par ailleurs: simvastatine, acétylsalicylate de DL-lysine, clopidogrel et bisoprolol. L´examen locomoteur retrouvait (Figure 1): 1) au membre inférieur gauche: une boiterie antalgique, un appui impossible sur la pointe du pied, une perte de l´équinisme physiologique du pied, une tuméfaction de la région postérieure de la cheville, une dépression douloureuse du relief achilléen. Le test de Thompson était positif; 2) au membre inférieur droit : une tuméfaction du relief achilléen qui était douloureuse à la dépression. Le test de Thompson était négatif.

**Figure 2:**
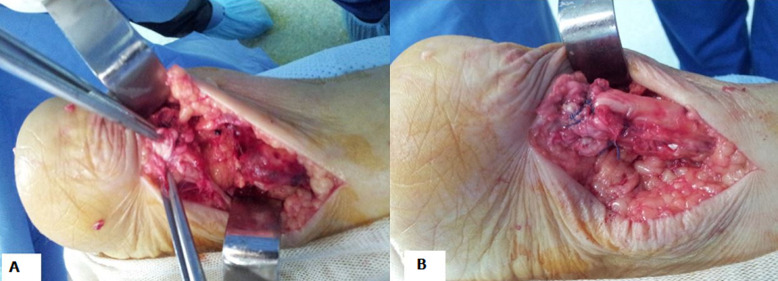
vue peropératoire: A) tendon d´Achille gauche rompu avec désorganisation des fibres tendineuses; B) réparation chirurgicale de la solution de continuité

Les radiographies standards étaient normales. L´échographie des chevilles montrait une rupture du tendon d´Achille gauche avec un épaississement des deux bouts respectifs, une fissuration achilléenne du tendon d´Achille droit associée à un épaississement de sa face profonde. Le diagnostic de tendinopathie achilléenne bilatérale compliquée d´une rupture du tendon d´Achille gauche était retenu. L´origine iatrogène liée à la prise de ciprofloxacine était suspectée justifiant l´arrêt de celle-ci. La responsabilité de la ciprofloxacine était estimée à l´aide de la méthode française d´imputabilité des effets inattendus ou toxiques des médicaments [3]. L´imputabilité intrinsèque était de niveau I2 (critère chronologique: 2; critère sémiologique: 2) et l´imputabilité extrinsèque de niveau B3. Biologiquement, il y avait un léger syndrome inflammatoire (protéine C réactive à 14 mg/l sans leucocytose), une anémie modérée normochrome normocytaire à 9 g/dl. La prise en charge était chirurgicale: réparation du tendon d´Achille rompu comme le montre la Figure 2. Les suites opératoires étaient simples et une rééducation fonctionnelle avait permis une bonne récupération locomotrice.

## Discussion

La fréquence des tendinopathies liées aux fluoroquinolones varie entre 15 et 20 accidents pour 100 000 sujets traités dont un tiers de cas sont compliqués de rupture tendineuse [1]. Les mécanismes exacts de l´atteinte tendineuse sont mal connus, la toxicité étant notée à des doses thérapeutiques [1]. Toutes les classes de fluoroquinolones sont incriminées, mais la péfloxacine et la ciprofloxacine occupent les premiers rangs [1,2,4,5]. La posologie de la ciprofloxacine per os conseillée chez l´hémodialysé varie de 250-750 mg/j après dialyse [6]. La dose prise par notre patiente était d´un gramme par jour soit largement supérieure aux posologies recommandées. L´incidence des effets indésirables associés aux fluoroquinolones est liée à l´âge, avec un âge de prédilection > 60 ans [1,2,4]. Cela laisse présager l´intervention d´un autre facteur de risque à part entière qui est le vieillissement tissulaire. Une prédominance masculine est soulignée mais, elle n´est pas absolue [1,4]. Le délai de survenu de la symptomatologie notée à cinq jours dans cette observation est compatible avec les données de la littérature [1,4,5,7]. Cependant, le délai maximal peut atteindre six mois. Tout porte à croire que le surdosage observé ici a peut-être contribué à la survenue rapide de cette complication. D´autres facteurs sont également reconnus. Hormis l´âge > 60 ans, nous avons recensé: l´insuffisance rénale chronique, l´hémodialyse, la corticothérapie et la prise d´une statine. L´insuffisance rénale chronique et l´hémodialyse interviendraient par le biais des troubles minéraux et osseux secondaires à l´hyperparathyroïdie. Les statines pourvues classiquement d´effets secondaires musculaires (rhabdomyolyse et myopathie), peuvent être en cause de tendinopathies. Les premiers cas en rapport avec les statines ont été publiés en 2001 [8]. Les corticoïdes constituent un facteur de risque quand ils sont pris par voie générale, inhalée ou locale directe. La présentation clinique de notre cas est classique. La douleur est le maître symptôme révélateur, associée à une impotence fonctionnelle. L´atteinte tendineuse intéresse majoritairement le tendon d´Achille, elle est bilatérale dans 40 à 66% des cas [1,5]. La rupture tendineuse en est la principale complication [5]. Elle expose à un handicap fonctionnel en l´absence de prise en charge.

## Conclusion

Notre observation fait état d´un effet secondaire rare mais, potentiellement grave lié aux fluoroquinolones, une classe de médicaments beaucoup utilisée en néphrologie. Cet effet secondaire expose à un handicap fonctionnel préjudiciable. D´autres facteurs favorisants connus sont: l´âge, l´insuffisance rénale chronique, l´hémodialyse chronique, la prise concomitante de statine ou de corticoïde. Les hémodialysés en plus d´avoir une hyperparathyroïdie et un retentissement cardiovasculaire, constituent par conséquent une population à risque comme c´est le cas dans cette observation. Par conséquent la communication pour le changement de comportement et la surveillance de ces patients même à distance d´un traitement par fluoroquinolone, revêt un intérêt non négligeable.
